# Layer-specific distribution and expression pattern of AMPA- and NMDA-type glutamate receptors in the barrel field of the adult rat somatosensory cortex: a quantitative electron microscopic analysis

**DOI:** 10.1093/cercor/bhac212

**Published:** 2022-06-21

**Authors:** Astrid Rollenhagen, Max Anstötz, Kerstin Zimmermann, Yu Kasugai, Kurt Sätzler, Elek Molnar, Francesco Ferraguti, Joachim H R Lübke

**Affiliations:** Institute of Neuroscience and Medicine INM-10, Research Centre Jülich GmbH, Leo Brandt Str., Jülich 52425, Germany; Institute of Neuroscience and Medicine INM-10, Research Centre Jülich GmbH, Leo Brandt Str., Jülich 52425, Germany; Institute of Anatomy II, Medical Faculty, University Hospital Düsseldorf, Heinrich-Heine-University, Universitätsstr. 1, Düsseldorf 40001, Germany; Institute of Neuroscience and Medicine INM-10, Research Centre Jülich GmbH, Leo Brandt Str., Jülich 52425, Germany; Department of Pharmacology, Medical University of Innsbruck, Peter Mayr Strasse 1a, Innsbruck A-6020, Austria; School of Biomedical Sciences, University of Ulster, Cromore Rd., Londonderry BT52 1SA, United Kingdom; School of Physiology, Pharmacology and Neuroscience, University of Bristol, University Walk, Bristol BS8 1TD, United Kingdom; Department of Pharmacology, Medical University of Innsbruck, Peter Mayr Strasse 1a, Innsbruck A-6020, Austria; Institute of Neuroscience and Medicine INM-10, Research Centre Jülich GmbH, Leo Brandt Str., Jülich 52425, Germany; Department of Psychiatry, Psychotherapy and Psychosomatics, RWTH/Medical University Aachen, Pauwelstr. 30, Aachen 52074, Germany; JARA Translational Medicine Jülich/Aachen, Germany

**Keywords:** neocortex, freeze fracture replication, postimmunogold-immunohistochemistry, quantitative electron microscopy, PSDs, glutamate receptor density maps

## Abstract

AMPA (α-amino-3-hydroxy-5-methyl-4-isoxazolepropionic acid) and NMDA (*N*-methyl-d-aspartate) glutamate receptors are driving forces for synaptic transmission and plasticity at neocortical synapses. However, their distribution pattern in the adult rat neocortex is largely unknown and was quantified using freeze fracture replication combined with postimmunogold-labeling. Both receptors were co-localized at layer (L)4 and L5 postsynaptic densities (PSDs). At L4 dendritic shaft and spine PSDs, the number of gold grains detecting AMPA was similar, whereas at L5 shaft PSDs AMPA-receptors outnumbered those on spine PSDs. Their number was significantly higher at L5 vs. L4 PSDs. At L4 and L5 dendritic shaft PSDs, the number of gold grains detecting GluN1 was ~2-fold higher than at spine PSDs. The number of gold grains detecting the GluN1-subunit was higher for both shaft and spine PSDs in L5 vs. L4. Both receptors showed a large variability in L4 and L5. A high correlation between the number of gold grains and PSD size for both receptors and targets was observed. Both receptors were distributed over the entire PSD but showed a layer- and target-specific distribution pattern.

The layer- and target-specific distribution of AMPA and GluN1 glutamate receptors partially contribute to the observed functional differences in synaptic transmission and plasticity in the neocortex.

## Introduction

At the molecular (subcellular) level, neurotransmitter receptors for the major excitatory and inhibitory systems are key elements controlling the “behavior” of synapses by regulating synaptic transmission, but also modulating plasticity (reviewed Greger and Esteban 2007; Hansen et al. 2007; Rao and Finkbeiner 2007). Moreover, it has been demonstrated that ionotropic glutamate receptors and their subunits are differentially expressed in excitatory principal neurons and GABAergic interneurons (see e.g. Baude et al. 1995; He et al. 1998; Matsubara et al. 1999; Nusser 1999; Takumi et al. 1999a, 1999b; Gonzalez-Albo and DeFelipe 2000; He et al. 2000; Nusser 2000; Kumar and Huguenard 2003; Matta et al. 2013; Kooijmans et al. 2014; Lalanne et al. 2016). Their differential expression and trafficking directly affects their targeting to and retention within synaptic compartments and thus, the magnitude of synaptic transmission (Dodt et al. 1998; Frick et al. 2001; Major et al. 2004a, 2004b; Kennedy and Ehlers 2006; Derkach et al. 2007; Lau and Zukin 2007; Shepherd and Huganir 2007; Kessels and Malinow 2009; Matta et al. 2013; reviewed by Newpher and Ehlers 2008). For example, the differential expression of AMPA-receptor subunits regulates both the deactivation/desensitization kinetics and Ca^2+^-permeability at principal neurons and GABAergic interneurons (Geiger et al. 1995; Geiger and Jonas 2000).

Paired recordings of various intra- and translaminar synaptic connections in the neocortex demonstrated co-localization of these receptors at synaptic contacts, but also described marked differences in the contribution of both AMPA and NMDA receptors to both excitatory and inhibitory synaptic transmission (see e.g. Markram 1997; Markram et al. 1997a, 1997b; Egger et al. 1999; Feldmeyer et al. 1999, 2002, 2006; Kumar and Ohana 2008; Marx and Feldmeyer 2013; Rollenhagen et al. 2015; Qi and Feldmeyer 2016; Seeman et al. 2018; reviewed by Lübke and Feldmeyer 2007; Feldmeyer 2010, 2012). Furthermore, there is growing evidence that the ability of the NMDA-receptor to either suppress or enhance synaptic transmission during spike-timing-dependent plasticity may critically depend on their pre- or postsynaptic location (Sjöstrom and Häusser 2006; Sjöstrom et al. 2007; Rodriguez-Moreno and Paulsen 2008).

Meanwhile, pre- and postembedding immunogold studies on ultrathin sections or on freeze fracture replica have been published describing the subcellular distribution and co-localization of AMPA- and NMDA-receptor subunits in the adult neocortex, hippocampus, and various other brain areas (e.g. Wenthold et al. 1996; Nusser 1999, 2000; Petralia et al. 1999, 2000, 2002; Lu et al. 2001; reviewed by Wenthold et al. 2003; Sheng and Hoogenraad 2007; Fukazawa and Shigemoto 2012).

However, for L4 of the somatosensory cortex, which is regarded to receive the majority of thalamocortical inputs (reviewed by Sherman 2012) and thus represents the first station of cortical information processing, and for L5, the major output system of the neocortex, rather little is known about the possible co-localization, density, and pre- or postsynaptic distribution of AMPA- and NMDA-type glutamate receptors at the ultrastructural (subcellular) level.

Here, we used freeze fracture replication (FFR) combined with single and/or double postimmunogold-labeling to detect their co-localization and to quantify their density and distribution pattern. AMPA- and NMDA-receptors were quantified separately at dendritic shaft and spine PSDs because the majority (~80–85%) are excitatory axo-spinous synaptic complexes in both L4 and L5; the remainder are shaft synaptic complexes in the adult rat barrel cortex. In addition, both structures represent different compartments at a given dendritic segment and are thus regarded to differentially contribute to synaptic transmission and plasticity (Choquet and Hosy 2020).

We demonstrate a layer- and target-specific difference in the density and distribution patterns of both receptors at L4 and L5 PSDs. However, individual PSDs showed significant differences in the density and ratio of AMPA vs. the GluN1-subunit of the NMDA-receptor.

Our findings may explain and partially contribute to the observed differences in functional properties of neocortical L4 and L5 excitatory synaptic connections in synaptic strength, efficacy, and short-term-plasticity (L4–L4: Feldmeyer et al. 1999; Egger et al. 1999, reviewed by Feldmeyer 2012; L5B–L5B: Markram et al. 1997a, 1997b; reviewed by Ramaswamy and Markram 2015), and may thus contribute to the stabilization, but also the layer-specific modulation of the columnar network.

## Material and methods

All experimental procedures were approved by the Animal Research Committee of the Research Centre Jülich GmbH, and complied with the guidelines laid out in the EU directive regarding the protection of animals used for experimental and scientific purposes (2004/23/EC).

### Cryosubstitution and FFR

In the neocortex, excitatory synaptic transmission is strongly mediated by AMPA- and NMDA-type glutamate receptors. To analyze and quantify their abundance, co-localization, density, and distribution pattern, single- or double postimmunogold-labeling was carried out on sodium lauryl sulfate (SDS)–FFR (Fujimoto 1995; reviewed by Harada and Shigemoto 2016). This method allows the visualization of the 2-dimensional distribution of integral membrane proteins retained by a carbon layer after solubilization of the tissue with SDS with high spatial resolution and high sensitivity.

For these experiments adult male Wistar rats (*n* = 15; ~3 months old; Charles River Laboratories, Sulzfeld, Germany) were deeply anesthetized with Narkodorm (60-mg/kg body weight) and then briefly (1 min) transcardially perfused with 0.1-M phosphate (PB)-buffered physiological saline (pH 7.4). This was followed by an ice-cold PB-buffered solution containing 1% paraformaldehyde and 15% of a saturated aqueous solution of picric acid for 12 min using a constant flow rotation pump (flow rate 8 mL/min; SCI 323, Watson-Marlow, Rommerskirchen, Germany). Brains were cut into 140-μm frontal sections with a Vibroslicer (Leica Microsystems VT 1000S, Vienna, Austria). Vibratome sections were then light microscopically inspected to identify the barrel field in the somatosensory cortex by the darker appearance of the barrels in L 4. Then squares (~2 × 2 mm) containing either L4 (including a barrel) or L5 (the area underneath the barrels) were trimmed, and cryoprotected with 30% glycerol overnight at 4°C. Sections were then high-pressure frozen (HPM 010; Bal-Tec, Balzers, Lichtenstein) and stored in liquid nitrogen before further use.

### Detergent-digested freeze fracture replica immunolabeling

Detergent-digested freeze fracture replica immunolabeling (FRIL) was performed according to published procedures (Kasugai et al. 2010). In brief, samples were fractured by freeze-etching (BAF 060; Bal-Tec, Balzers, Lichtenstein). Fractured faces were replicated by evaporation of carbon (rotating) by means of an electron beam gun positioned at a 90° angle to a thickness of 5 nm and shadowed unidirectionally with platinum-carbon at a 60° angle (thickness 2 nm). Finally, a 15-nm thick layer of carbon was applied from a 90° angle (rotating). Tissue was solubilized in a solution containing 2.5% SDS and 20% sucrose made up in 15-mM Tris-buffered saline (TBS, pH 8.3) on a shaking platform for 18 h at 80°C. Replicas were stored in the same solution at room temperature until further processed for postimmunogold-immunohistochemistry.

### Postimmunogold-immunohistochemistry on detergent-digested freeze fracture replica

Before incubation in the primary antibodies, replicas were rinsed in 50-mM TBS containing 2.5% bovine serum albumin (BSA, Fraction V, Sigma A9647, Munich, Germany) and 0.05% sodium azide (pH 7.4) for 5 min. This was followed by 50-mM TBS (3 × 10 min each) and blocking for unspecific binding in the same buffer containing 5% BSA (Sigma, Munich, Germany) for 1 h. FFRs were then transferred to droplets (30 μL) of the primary antibody (self-raised rabbit panAMPA polyclonal antibody recognizing all 4 GluR1–4 subunits, 1:250; provided by Prof. Elek Molnar, University of Bristol; mouse GluN1 monoclonal antibody cat. no. MAB363, 1:300, Millipore-Merck, Darmstadt, Germany) diluted in 50-mM TBS containing 1.25% BSA for 48 h in a wet chamber. The 2 antibodies were raised against epitopes in the extracellular domain (N-terminus). After washing in 50-mM TBS (3 × 10 min), FFRs were incubated overnight at 4°C in a solution containing the appropriate immunogold-conjugated secondary antibody (British Biocell Intern. Ltd, Cardiff, United Kingdom) diluted in the same buffer as used for the primary antibodies. For the detection of either AMPA (goat anti-rabbit IgG, 1:30) or GluN1 (goat-anti-mouse IgG, 1:30) and the subsequent quantitative analysis of gold particle distributions, single labeling was carried out using 5 nm (goat-anti-rabbit IgG product code: EM.GAR5, batch number: 15,266; goat-anti-mouse IgG: product code: EM.GMHL5, batch number 16,035) sized gold particles. For the co-localization of AMPA- and the GluN1-subunit of the NMDA-receptor, a mixture of both primary antibodies was used. For the secondary gold-conjugated antibodies a combination of either 5/10 nm (goat-anti-rabbit IgG product code: EM.GAR10, batch number: 11,268; goat-anti-mouse IgG: product code: EM.GMHL10, batch number: 15,329) or 10/15 nm (goat-anti-rabbit IgG product code: EM.GAR15, batch number: 15,495; goat-anti-mouse IgG: product code: EM.GMHL15, batch number: 14,754) sized gold particles was used. Finally, FFRs were washed thoroughly in 50-mM TBS (3 × 10 min) and then in purified double distilled water (2 × 5 min). Finally, they were then transferred and mounted onto pioloform-coated parallel line copper grids (Plano GmbH, Wetzlar, Germany), air-dried and stored in grid boxes until electron microscopic (EM) examination. All experiment if not stated otherwise were carried out at room temperature.

### EM examination and quantitative analysis of freeze fracture replica immunolabeling

FRILs were examined using a Zeiss Libra 120 transmission EM (Carl Zeiss, Oberkochen, Germany) equipped with a bottom mounted 2K Proscan digital camera (Tröndle, Moorenweis, Germany). For general documentation, digital images were taken using the SIS analysis software (Olympus GmbH, Hamburg, Germany) and stored as TIFF-files in a database until further use. For the quantitative analysis, only PSDs at dendritic shaft and dendritic spine synapses showing no signs of disruption, malformation, or distortions were taken and photographed at a primary magnification of ×25.000. These EM images provided the basis for the subsequent quantitative analysis and were imported into the software OpenCAR (Sätzler et al. 2002) and further analyzed as described in detail below.

### Quantitative analysis of receptor density and distribution using OpenCAR

**Fig. 1:**
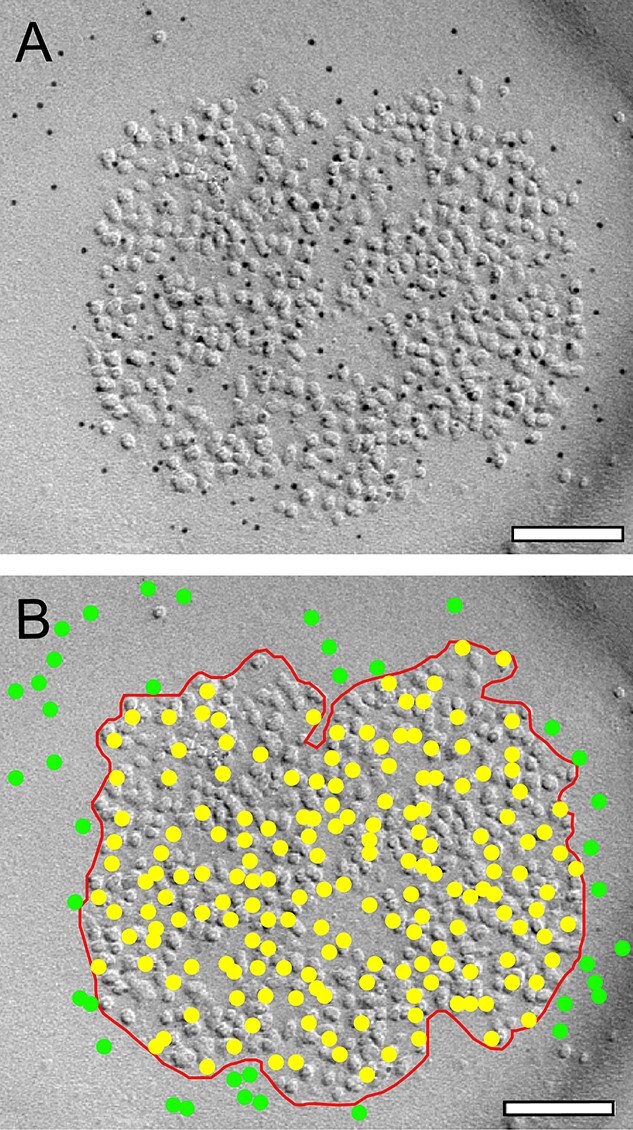
Quantitative analysis of the density and distribution pattern of gold particles at individual PSDs. A) High power electron micrograph showing the intrasynaptic distribution of the AMPA receptor at a shaft PSD in L4 of the adult rat “barrel” cortex (indicated by the 5-nm gold particles), visible as a cluster of intramembranous particles on the exoplasmic face of a replica, using SDS-FRIL. B) Same image as in A). Here, a contour line (red) is drawn to outline the PSD to estimate its surface area. Individual gold particles within the PSD contour are highlighted as yellow dots, extra-synaptic gold particles in green that were neglected in the quantitative analysis. Scale bar in A), B 0.1 μm.

For the quantitative analyses, TIFF-images were imported into OpenCAR. PSDs were sorted for individual animals, layers (L4 vs. L5) and target locations (dendritic shaft vs. dendritic spine PSDs). First, in all digital images, the PSD was defined by the dense accumulation of intramembrane particles (IMPs; Kasugai et al. 2010; Fig. 1A). A contour line was then drawn using the most outer IMPs defining the border of the PSDs (Fig. 1B) that was done by 2 independent observers, which came to similar results. These contours allowed the measurement of the PSD surface area using OpenCAR. Then the number of gold grains (yellow dots in Fig. 1B) was counted within the contoured area. Gold grains outside the contour line (green dots) were neglected due to their extra-synaptic locations and were thus not included in the analysis (Fig. 1B).

### Generation of receptor density maps

For the generation of receptor distribution maps, a previously published approach (Kasugai et al. 2006) with some modifications was used. To this purpose a custom script written in Visual Basic was developed. After contouring the PSD and marking the position of gold grains (Fig. 1B and Supplemental Fig. 1A), the center of gravity was calculated for the PSD contour (Supplemental Fig. 1B). Next, the maximal diameter (also called maximum Ferets diameter) of the PSD was determined (Supplemental Fig. 1C) and the PSD rotated such to orient the maximal diameter to the horizontal axis (Supplemental Fig. 1D). Then, a Cartesian grid (25 by 25 nm) was placed over the reconstructed PSD (Supplemental Fig. 1E) and finally the number of gold grains/markers in each grid (indicated by different colored squares) was counted. This results in a receptor density map for individual PSDs (Supplemental Fig. 1F). Then, an average of all individual density maps was created. Finally, a calculation of area values was achieved by summating the area of grid segments that are enclosed by their respective PSD contour. To account for variability in size and shape of the PSDs, the average density map was normalized by dividing every density/grid segment value by its related area value.

### Radial distribution-analysis of the distribution pattern of gold grains

As for the density plots, reconstructed PSDs were imported to a custom script written in Visual Basic. The distances of every gold grain/marker to the center of the PSD were calculated. The obtained distances were binned and counted in 20-nm segments. As described previously, to account for variability in size and shape of the PSDs, the values of each segment were normalized by dividing them with its related area value. For the Radial distribution analysis, the measured values were compared with simulated distributions (see below) using a repeated measures 2-way analysis of variance (ANOVA) on ranks with Dunnet post-hoc test.

### Generation of receptor density maps

For the generation of receptor distribution maps, a previously published approach (Kasugai et al. 2006) with some modifications was used. To this purpose a custom script written in Visual Basic was developed. After contouring the PSD and marking the position of gold grains (Fig. 1B and Supplemental Fig. 1A), the center of gravity was calculated for the PSD contour (Supplemental Fig. 1B). Next, the maximal diameter (also called maximum Ferets diameter) of the PSD was determined (Supplemental Fig. 1C) and the PSD rotated such to orient the maximal diameter to the horizontal axis (Supplemental Fig. 1D). Then, a Cartesian grid (25 by 25 nm) was placed over the reconstructed PSD (Supplemental Fig. 1E) and finally the number of gold grains/markers in each grid (indicated by different colored squares) was counted. This results in a receptor density map for individual PSDs (Supplemental Fig. 1F). Then, an average of all individual density maps was created. Finally, a calculation of area values was achieved by summating the area of grid segments that are enclosed by their respective PSD contour. To account for variability in size and shape of the PSDs, the average density map was normalized by dividing every density/grid segment value by its related area value.

### Simulation of different receptor distributions

To simulate different distributions of gold grains for each receptor, the reconstructed PSDs and number of markers were kept, but the position values of markers were replaced by randomly generated values. As a result, 3 different types of simulated distributions of markers were generated: an equal distribution, a Gaussian distribution with its peak at the center, and a Gaussian distribution with its peak at the contour/border of the PSD. For the random equal distribution, a random number generator (Bains 2008) was used to create a value for the horizontal (*x*) and the vertical (*y*) position of a marker (within the PSD outline). A similar procedure was performed for the center and border-Gaussian distribution, but with a higher probability that a marker is placed at the center or border (related to the distance of a marker to the center of the PSD), respectively. As a result, the random marker placements followed the probability density function of a Gaussian normal distribution (Supplemental Fig. 2). The simulation was iterated 100 times for each individual PSD and results were averaged. This resulted in a dataset containing samples paired with simulations.

### General statistics

Statistical testing for multiple groups was either performed using a H-test with post-hoc Mann–Whitney *U*-test (GraphPad Prism, GraphPad Software Inc., CA, United States) or with a Mann–Whitney *U*-test comparing 2 groups using OriginPro 2020b (OriginLab Corp. MA, United States). Statistical comparison of radial distributions, comparing samples vs. simulations was performed using a 2-way repeated measures ANNOVA on ranks with Dunnett post-hoc testing.

The level of significance was set to *P* < 0.05. Values in the manuscript are given as mean ± standard deviation (SD). The mean value is expressed as the total mean over single means ± SD. In addition the median with the 1st and 3rd quartile (Interquartile Range, IQR), the coefficient of correlation (*R*^2^), the coefficient of variation (CV), the skewness, the degree of asymmetry observed in a probability distribution, and the variance, a statistical measure of variability, was given for each structural parameter analyzed.

## Results

Here the co-localization, density, and distribution pattern of the AMPA-receptor (GluR1–4) and the GluN1-subunit of the NMDA-receptor was investigated by means of FRIL at L4 and L5 synaptic complexes in the barrel field of the adult rat somatosensory neocortex.

A total of 70 PSDs at dendritic shaft and spine synapses showing no distortions, disruptions and/or malformations were examined for the co-localization of both receptors in L4 (*n* = 30 PSDs) and L5 (*n* = 40 PSDs). For the quantitative analysis of receptor density and distribution pattern, a total of 1,429 PSDs located on dendritic shafts and spines were examined.

### Co-localization of AMPA- and GluN1 at cortical L4 and L5 synaptic complexes

**Fig. 2:**
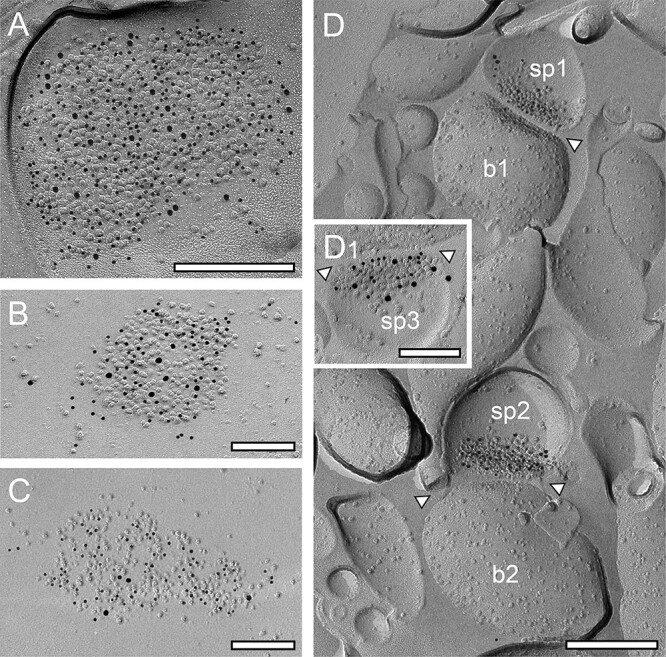
Co-localization of AMPA-receptors and the GluN1-subunit of the NMDA-receptor at cortical L4 and L5 dendritic shaft and spine PSDs. A, B) Two dendritic shaft PSDs in L4, a large one A) with a high density of gold grains detecting AMPA receptors (GluR1–4, represented by 5-nm gold particles) co-localized with gold grains detecting GluN1-subunit of the NMDA-receptor (10-nm gold particles) and a comparably smaller PSD B) with a lower density in both gold grains detecting AMPA- and GluN1. Scale bar in A) 0.25 μm and B) 0.1 μm, respectively. C) Distribution of AMPA-receptors (5-nm gold particles) and the GluN1 (10-nm gold particles) at a somatic PSD of a neuron located in L5. Scale bar 0.1 μm. D) Two PSDs (sp1-sp3) at spine synapses (b1 and b2) in L4 where AMPA receptors (5-nm gold particles) and GluN1 (10-nm gold particles) are co-localized, but with different densities and distribution patterns. Scale bar C 0.25 μm. D1) Higher magnification of another spine PSD in L4 also showing co-localization of AMPA (5-nm gold particles) and GluN1 (10-nm gold particles). Note the differences in the density and distribution pattern of both receptors at individual PSDs. The synaptic cleft in D) and D1) is indicated by arrowheads. Scale bar 0.1 μm.

The majority of L4 (~95%) and L5 dendritic shaft and spine PSDs (~95%) contained both AMPA and the GluN1-subunit of the NMDA-receptor, only in a few cases pure AMPA-receptor or GluN1-subunit receptor containing dendritic shaft or spine PSDs were observed. Co-localization of both receptors was found on dendritic shafts (Fig. 2A and B), different types of spines (Fig. 2C and C1) and somata (Fig. 2E) at L4 and L5 PSDs.

**Fig. 3:**
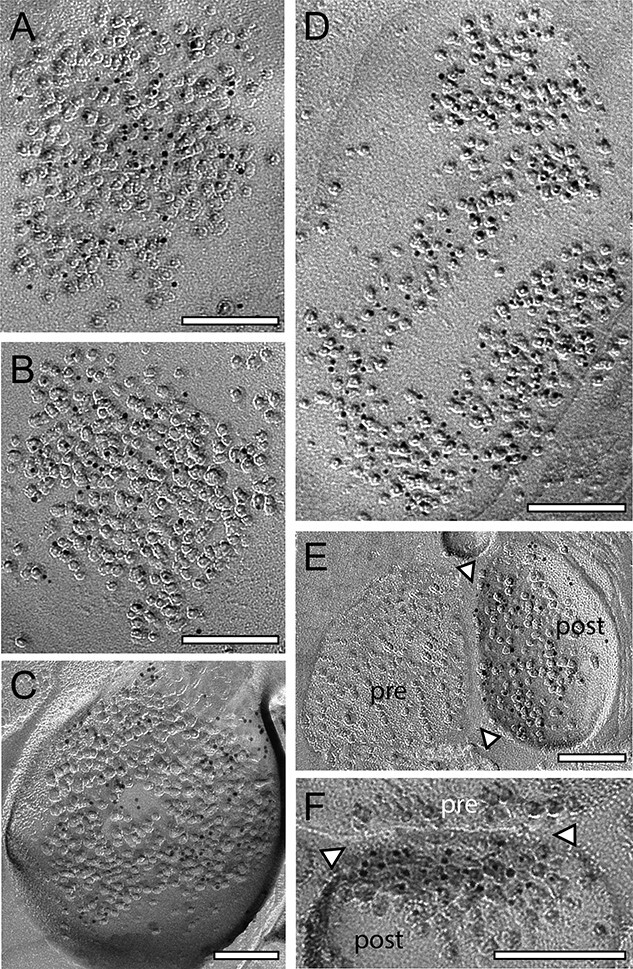
Density and distribution patterns of AMPA receptors and the GluN1-subunit of the NMDA-receptor at dendritic shaft and spine PSDs at L4 synaptic contacts. A–B) Two examples of 2 large shaft PSDs with a macular, non-perforated appearance labeled with gold grains detecting for AMPA- A) and the GluN1 B). C) Ring-like spine PSD labeled with gold grains detecting the GluN1. D) Large dendritic shaft PSD with a horseshoe to ring-like appearance labeled with gold grains detecting AMPA receptors. E, F) Two spine PSDs with a comparably low density of gold grains detecting AMPA E) and the GluN1 F). In both images, the synaptic cleft is marked by arrowheads. *Abbreviations*: pre: presynaptic; post: postsynaptic. Scale bars in A–F) 0.1 μm.

**Fig. 4:**
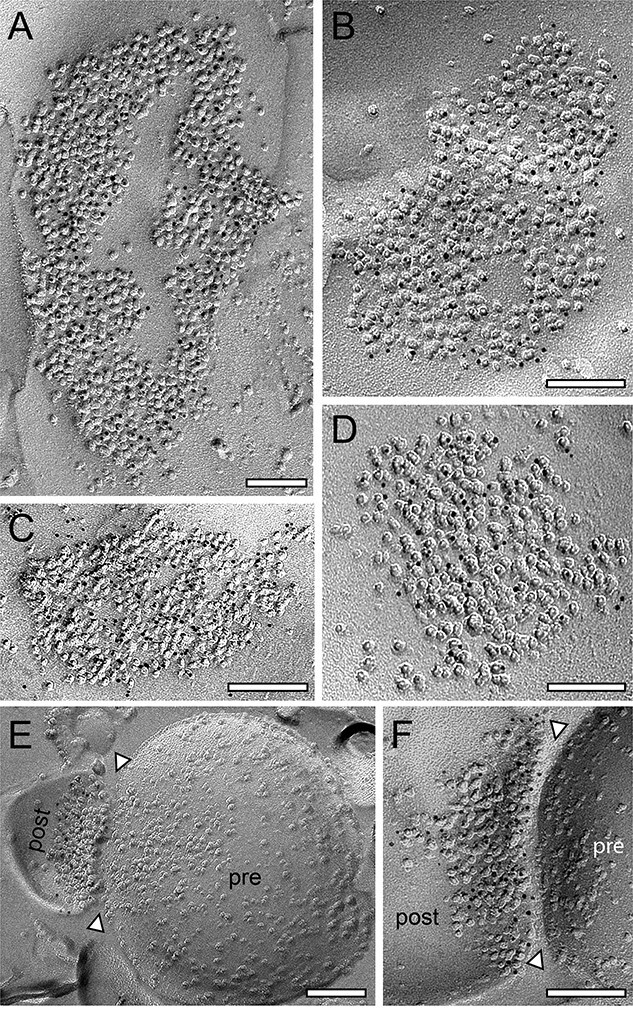
Density and distribution patterns of AMPA receptors and the GluN1-subunit of the NMDA-receptor at dendritic shaft and spine PSDs at L5 synaptic contacts. A, B) Two large dendritic shafts PSDs with a ring-like A) and somewhat perforated B) appearance labeled with gold grains detecting AMPA receptors. C, D) Two examples of macular, non-perforated dendritic shaft PSDs with gold grains detecting GluN1. E, E1) Small spine PSD with a comparably low density of gold grains detecting the AMPA receptors. The synaptic cleft is marked by arrowheads. F) High magnification of a spine PSD labeled with gold grains detecting the GluN1. The synaptic cleft is marked by arrowheads. Scale bar in A–F) 0.1 μm.

Dendritic shaft and spine PSDs, in both L4 and L5, displayed different morphological features. The majority (~80%) were round to oval shaped, non-perforated, and macular PSDs (Figs. 2A, B, 3C, and 4D), the remaining were either perforated (~10%; Figs. 3A, 4B, and C), horseshoe- (~5%; Fig. 3B), or ring-like (~5%; Fig. 4A). On spine PSDs, also non-perforated macular (Figs. 2C, D, 3E, and 4E, F), and perforated ring-like types (Figs. 3D, E, and 4E1) were found with a similar ratio as observed on dendritic shafts. However, PSDs on both target structures displayed a great variability in the shape and size of PSDs in L4 and L5.

### PSD surface areas on dendritic shafts and spines in L4 and L5

**Fig. 5:**
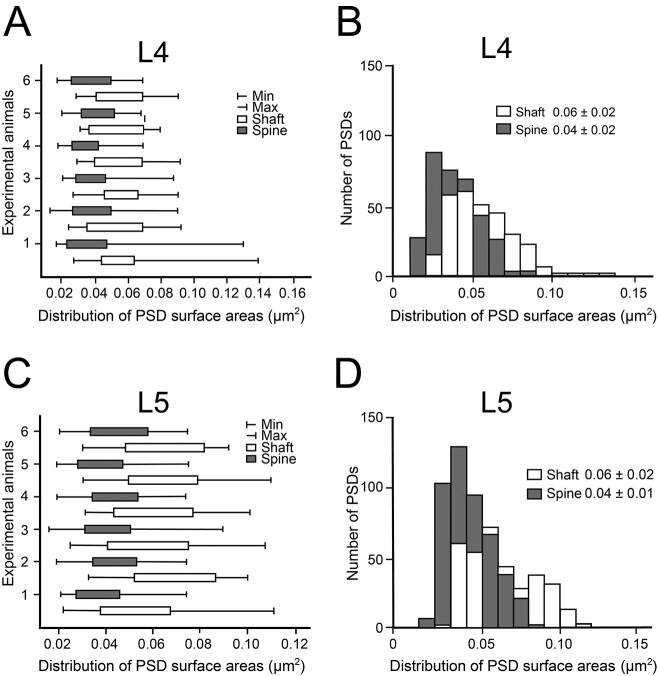
Distribution of PSD surface areas at L4 and L5 synaptic complexes. A, C) Box plots showing the distribution of PSD surface areas between experimental animals for L4 A) and L5 C). Each bar represents the distribution of PSD surface area for the individual experimental animals investigated separated for dendritic shaft (in white) and spine (in gray) PSDs. B, D) Bar histograms showing the distribution of PSD surface areas separated for shaft (in white) and spine (in gray) PSDs at synaptic complexes in L4 B) and L5 D).

To correlate the density of both receptors, the surface area for all PSDs (*n* = 1,429) that contained gold grains (Fig. 5) was determined by generating a contour around the PSD (Fig. 1B). The quantitative analysis of the distribution of PSD surface areas was performed for L4 (Fig. 5A and B) and L5 (Fig. 5C and D) and was separated for shaft and spine PSDs.

In both cortical layers dendritic shaft PSDs were significantly larger (*P* < 0.001) when compared with spine PSDs, although a great variability in PSD size was observed for both target structures (ranging from ~ 0.01 μm^2^, see e.g. Fig. 2B and C1, to ~0.16 μm^2^, see e.g. Figs. 3B and 4A) and layers as indicated by the minimum and maximum values, skewness and variance (Fig. 5; Table 1). On average, dendritic shaft PSDs were ~1.5-fold larger than spine PSDs (Fig. 5B and D) in both L4 and L5 (L4: dendritic shaft PSDs 0.06 ± 0.02 μm^2^ and spine PSDs 0.04 ± 0.02 μm^2^; L5: dendritic shaft PSDs 0.06 ± 0.02 μm^2^ and spine PSDs 0.04 ± 0.01 μm^2^).

**Table 1:** Quantitative analysis of the distribution of PSD surface areas at L4 and L5 dendritic shafts and spines.

	**L4**		**L5**	
	**Dendritic shaft**	**Dendritic spine**	**Dendritic shaft**	**Dendritic spine**
**PSD surface area [μm** ^**2**^**]**				
No. of PSDs	293	337	335	463
Mean ± SD	0.06 ± 0.02^***^	0.04 ± 0.02	0.06 ± 0.02^***^	0.04 ± 0.01
Median; IQR	0.05; 0.03	0.04; 0.02	0.06; 0.04	0.03; 0.02
Min	0.02	0.01	0.02	0.02
Max	0.15	0.13	0.16	0.08
Skewness	0.85	−1.08	0.63	0.54
Variance	0.000381131	0.00402364	0.00048223	0.00020761
CV	0.33	0.50	0.33	0.25

Dendritic shaft vs. dendritic spine PSDs in both L4 and L5.

^***^ *P* < 0.001; Graph Pad Prism H-test with post-hoc Mann–Whitney *U*-test.

### Density and distribution pattern of AMPA- and GluN1 receptors in L4 and L5 synaptic complexes

The quantitative analysis of the density and distribution pattern of both receptors in L4 and L5 was performed using 5-nm sized gold grains (Figs. 3 and 5). Either the number of gold grains/PSD or gold grains/μm^2^ (Table 2) is given. In L4 118 dendritic shaft, 182 spine PSDs for the AMPA-receptor, 175 dendritic shaft, 155 spine PSD for the GluN1-subunit of the NMDA-receptor, and in L5 166 dendritic shaft, 281 spine PSD for the AMPA-receptor and 169 dendritic shaft and 182 spine PSDs for the GluN1-subunit of the NMDA-receptor were analyzed.

**Table 2:** AMPA- and GluN1 receptor density at L4 and L5 PSDs in the rat somatosensory neocortex.

	**L4**		**L5**	
	**Dendritic shaft**	**Dendritic spine**	**Dendritic shaft**	**Dendritic spine**
**Receptor density gold grains/PSD**				
**AMPA**				
No. of PSDs	118	182	166	281
Mean ± SD	22.70 ± 13.73	24.02 ± 13.38	37.02 ± 21.58	30.29 ± 15.58
Median; IQR	20.00¸ 18.25	22.00; 14.25	33.50; 25.00	27.00; 20.00
Min	4	4	5	3
Max	71	76	131	103
Skewness	1.22	1.51	1.37	1.20
Variance	188.47	179.02	465.64	242.83
CV	0.60	0.56	0.58	0.51
**GluN1**				
No. of PSDs	175	155	169	182
Mean ± SD	30.19 ± 17.85	15.43 ± 9.04	39.23 ± 24.02	21.67 ± 13.07
Median; IQR	27.00; 24.00	14.00; 11.00	35.00; 31.00	19.00; 15.00
Min	5	3	5	2
Max	82	47	139	82
Skewness	0.90	1.24	1.34	1.61
Variance	318.59	81.77	576.76	170.76
CV	0.59	0.59	0.61	0.60
**Receptor density gold grains/μm** ^**2**^				
**AMPA**				
Mean ± SD	416 ± 214	653 ± 329	634 ± 282	769 ± 284
Median; IQR	390; 306	600; 443	601; 394	756; 348
Min	79	171	46	108
Max	1,083	1,777	1,421	1,691
Skewness	0.81	0.80	0.30	0.23
Variance	4.59	10.84	7.94	8.09
CV	0.51	0.50	0.44	0.37
**GluN1**				
No. of PSDs	175	155	169	182
Mean ± SD	557 ± 264	429 ± 231	622 ± 300	511 ± 287
Median; IQR	539; 425	382; 285	605; 396	447; 392
Min	101	78	98	29
Max	1,352	1,369	1,472	13.12
Skewness	0.32	1.00	0.45	0.81
Variance	6.98	5.32	9.02	8.26
CV	0.47	0.54	0.48	0.56

Despite L4 AMPA dendritic shaft PSDs vs. L4 AMPA spine PSDs, L4 AMPA dendritic shaft PSDs vs. L5 GluN1 spine PSDs, L4 GluN1 dendritic shaft PSDs vs. L5 AMPA spine PSDs, and L5 AMPA dendritic shaft PSDs vs. L5 GluN1 dendritic shaft PSDs all other values tested were significantly different using a Graph Pad Prism H-test with post-hoc Mann–Whitney *U*-test with ^***^P < 0.001 and ^**^P < 0.01, respectively.

**Fig. 6:**
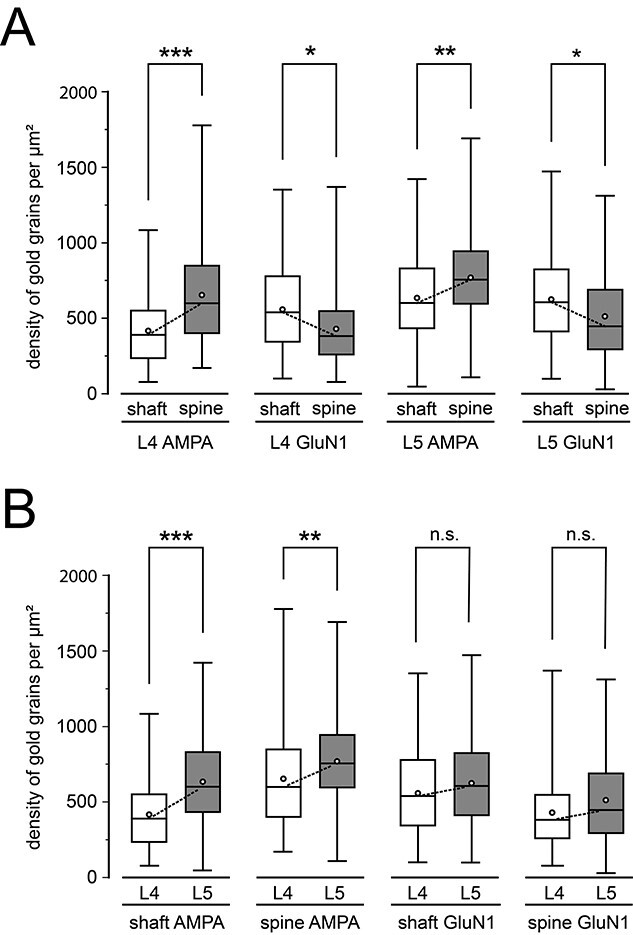
Quantitative analysis of the receptor distribution at L4 and L5 synaptic complexes. A) Box plot of receptor densities detected by immuno-labeled gold grains comparing shaft vs. spine PSDs in the same layer and receptor type. B) Box plots of receptor densities but comparing the 2 target layers and receptors in both layers. In all box plots, the length of the box represents the upper and lower quartile, the bar represents the median, the open circles the mean and the lines indicate the minimum and maximum receptor density. Dashed lines indicate the comparison between the corresponding medians. Significant differences were tested with a ranked 2-way ANOVA and post-hoc Fisher’s LSD test. ^***^P < 0.001; ^**^P < 0.01; and ^*^P < 0.05; n.s.: non-significant.

In L4 the number of gold grains/PSD detecting the AMPA-receptor was similar between dendritic shafts vs. spines (22.70 ± 13.73 vs. 24.02 ± 13.38) with a minimum of 0.79 and a maximum of 10.83 (Table 2 and Fig. 6). In contrast, the number of gold grains detecting the AMPA-receptor/μm^2^ on spines significantly (*P* < 0.001) exceeds that on dendritic shafts by nearly 1.6-fold. In contrast, for the GluN1-subunit of the NMDA-receptor a marked and significant difference (*P* < 0.001) by ~2-fold (30.19 ± 17.85 vs. 15.43 ± 9.04) in favor of dendritic shaft vs. spine PSDs in L4 was observed for the receptor density/PSD (see also Fig. 6) whereas using the gold grains/μm^2^ criterion this difference was nearly 1.3-fold larger on dendritic shafts vs. spines (Table 2). In L5, the number of gold grains/PSD detecting the AMPA-receptor was by ~1.2-fold higher but non-significant on dendritic shaft PSDs when compared with that on spines (37.02 ± 21.58 vs. 30.29 ± 15.58; see also Table 2) and also larger by ~1.2-fold when using the number of gold grains/μm^2^. For the GluN1-subunit of the NMDA-receptor the number of gold grains/PSD was significantly higher (*P* < 0.001) by ~1.8-fold (39.23 ± 24.02 vs. 21.67 ± 13.07) on dendritic shafts than on spines (Fig. 6A) and by ~1.2-fold higher on dendritic shafts vs. spines using the number of gold grains/μm^2^.

A significant (*P* < 0.001) higher number of gold grains detecting the AMPA-receptor/PSD was observed at both dendritic shaft (37.02 ± 21.58 vs. 22.70 ± 13.73) and spine PSDs (30.29 ± 15.58 vs. 24.02 ± 13.38) in L5 vs. L4 PSDs by ~1.6 and ~1.3-fold, respectively. By using the number of gold grains/μm^2^ the difference was ~ 1.7 and ~1.2-fold for both target structures in favor of L5 vs. L4. A significant difference (*P* < 0.001) was found for the GluN1-subunit of the NMDA-receptor between the two cortical layers by ~1.3-fold for L5 vs. L4 dendritic shaft PSDs (39.23 ± 24.02 vs. 30.19 ± 17.85) and ~1.4-fold for L5 vs. L4 spine PSDs. This difference was minor for the gold grains/μm^2^ criterion by ~1.1-fold for both target structures also in favor of L5 vs. L4 (Table 2).

**Fig. 7:**
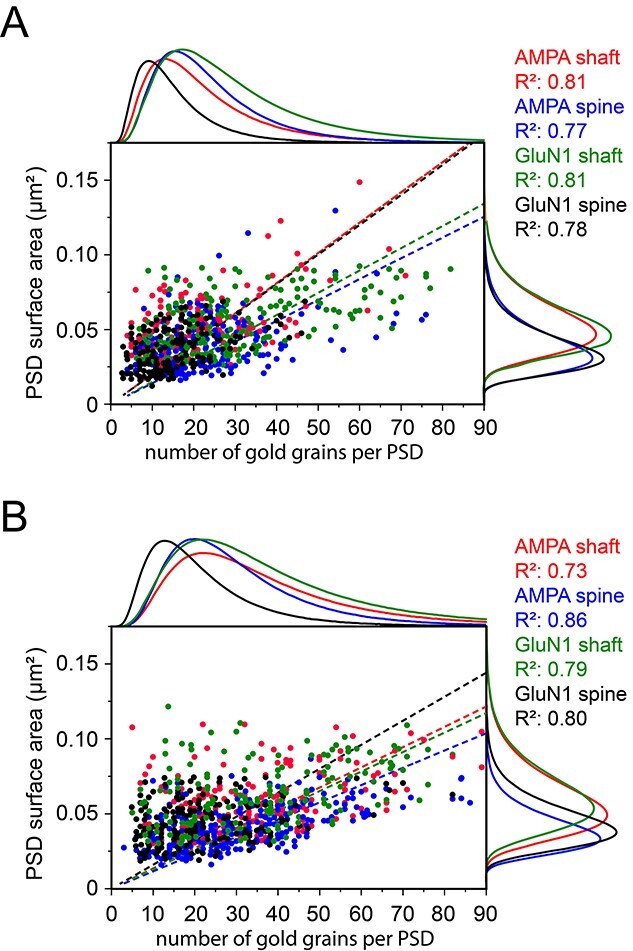
Correlation of gold grains detecting AMPA receptors and the GluN1-subunit of the NMDA-receptor with PSD surface areas at L4 and L5 synaptic complexes. A, B) Dot plots of all analyzed PSDs illustrating the number of gold grains plotted against the PSD surface area for L4 A) and L5 B). For better illustration, the distribution of each receptor, its target structure and the *R*^2^-values are given in different colors. Adjacent histograms illustrate the distribution of data points at the *x*- and *y*-axis. Note the high correlation between PSD surface area with receptor density in both cortical layers.

In summary, in L4 the number of gold grains/PSD or per μm^2^ detecting the AMPA-receptor was highest on spines (*P* < 0.001), followed by the GluN1-subunit of the NMDA receptor on dendritic shafts (*P* < 0.001), and with lower numbers for the AMPA-receptor on dendritic shafts and the GluN1-subunit of the NMDA receptor on spines but with no significant differences (Fig. 7A1). In L5, the number of gold grains/PSD and per μm^2^ was also highest for the AMPA-receptors on spines (*P* < 0.001), but quite similar for the AMPA-receptors on dendritic shafts and GluN1-subunit of the NMDA-receptor on spines and lowest for GluN1-subunit of the NMDA-receptor on L5 spines (Fig. 7B1). It has to be noted that all receptors showed a large variability in both layers as indicated by the SD, skewness, variance, and CV (Fig. 6; Table 2).

In general, both receptors showed a high correlation with PSD size in L4 and L5 (L4: Fig. 7A, L5: 7B) with highest *R*^2^-values for the AMPA-receptor and GluN1-subunit of the NMDA receptor on L4 dendritic shafts (0.81), but only slightly lower values for the AMPA-receptor and GluN1-subunit of the NMDA-receptor on L4 spines (0.77 and 0.78). In L5, *R*^2^-values were highest for the AMPA-receptor and GluN1-subunit of the NMDA-receptor on spines (0.86 and 0.80), nearly similar for the GluN1-subunit of the NMDA-receptor on dendritic shafts (0.79) and lowest for the AMPA-receptor on dendritic shafts (0.73).

### AMPA/NMDA ratio at L4 and L4 PSDs

**Fig. 8:**
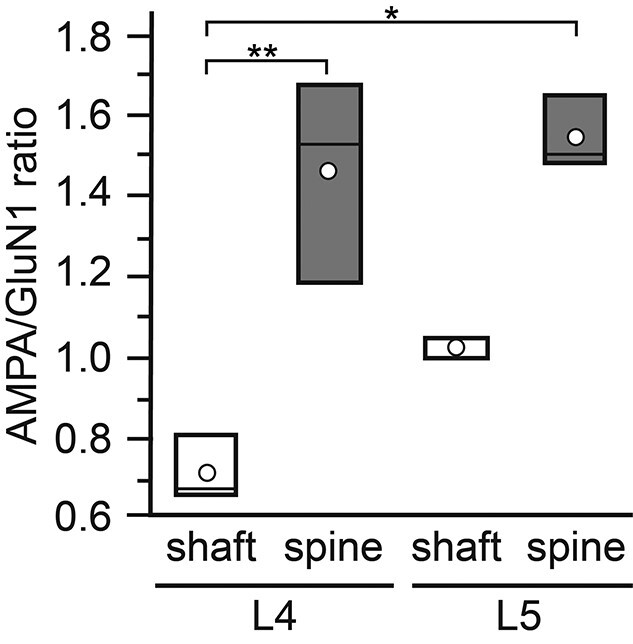
Bar histogram showing the AMPA/GluN1 ratio at L4 and L5 PSDs. AMPA/GluN1 ratio in L4 and L5 separated for dendritic shaft (open bars) and spine (gray bars) PSDs. In all bars, the length of the box represents the upper and lower quartile, the line the median, and the open circle the mean. Note that the values for L4 and L5 spine PSDs are quite similar but much higher when compared with those for dendritic shaft PSDs with lowest values for L4 dendritic shaft PSDs. ^*^P < 0.05; ^**^P < 0.01 (Graph Pad Prism H-test with post-hoc Mann–Whitney *U*-test).

To better understand the relationship between AMPA-receptors and the GluN1-subunit of the NMDA- receptor, a layer- and target-specific ratio analysis was performed (Fig. 8). Strikingly, the two highest average ratio values were found for L4 spine (1.46 ± 0.25) and L5 spine PSDs (1.54 ± 0.09) with a lower value for L5 dendritic shaft (1.03 ± 0.02) and the lowest for L4 dendritic shaft PSDs (0.72 ± 0.08). The average mean value for L4 spine PSDs was ~2-fold larger than that of L4 dendritic shaft PSDs whereas that for L5 spine vs. L5 dendritic shaft PSDs was ~1.5-fold. Significant differences were found for L4 dendritic shaft vs. L4 spine (*P* < 0.01) and L4 dendritic shaft vs. L5 spine PSDs (*P* < 0.05). The AMPA/GluN1 ratio analysis demonstrated that spine PSDs in L4 and L5 contained the highest density of AMPA-receptors whereas at dendritic shaft PSDs either an equal ratio between AMPA/GluN1 (L5) or a ratio in favor of the GluN1-subunit of the NMDA-receptor was observed.

These results suggest that AMPA-receptors guarantee a fast and reliable induction of the EPSP and contribute with a large fraction to the overall EPSP amplitude at L4 and L5 spine PSDs whereas the GluN1-subunit of the NMDA-receptor due to the elimination of the Mg^2+^-block may contribute to a prolonged time course of the EPSP thereby modulating short-term plasticity. The high density of dendritic spines at L4 excitatory spiny neurons and L5 pyramidal cells together with the high density of AMPA-receptors at spines suggest that these structures may act as coincidence detectors (see also Discussion).

### Receptor density maps of AMPA-receptors and the GluN1-subunit of the NMDA-receptor in L4 and L5 of the adult rat somatosensory neocortex

**Fig. 9:**
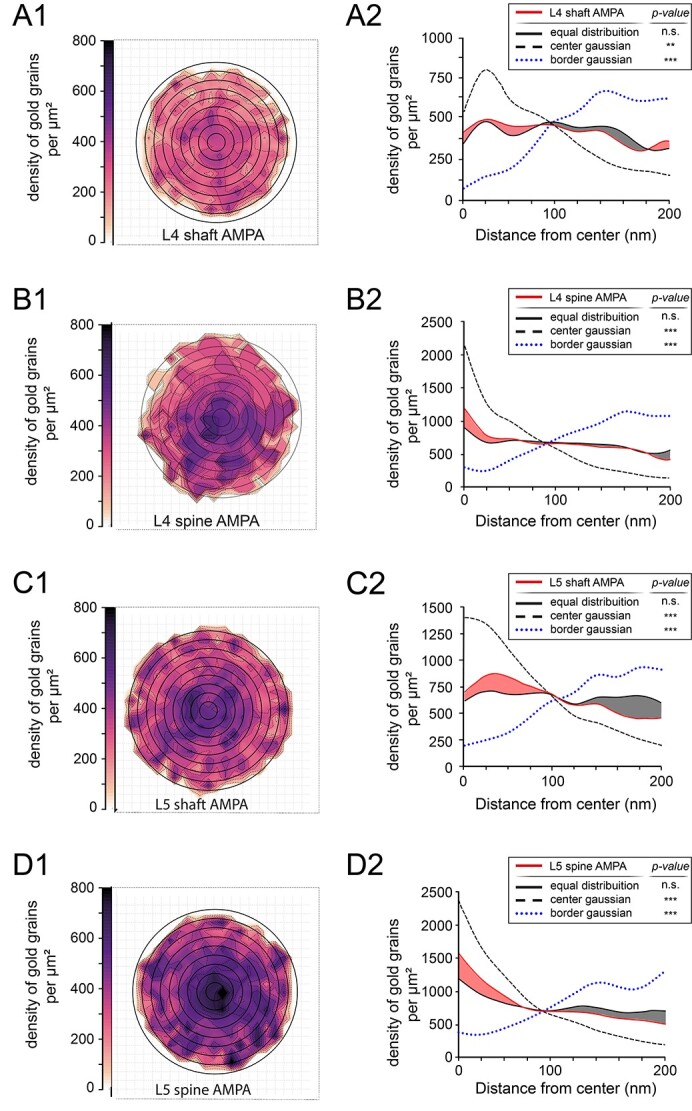
AMPA-receptor distribution pattern at L4 and L5 PSDs. A1) Two-dimensional density plot illustrating the spatial distribution of gold grains detecting the AMPA-receptors at L4 dendritic shaft PSDs. Grid in the background indicates the 20 by 20-nm binning of analysis. Note the homogeneous intensity pattern suggesting an equal distribution of receptor labeling detected by gold grains. A2) One-dimensional radial distribution-analysis illustrating the density of gold grains for the actual/measured PSDs (solid red line), a simulated equal distribution (solid black line) at L4 dendritic shaft PSDs. Colored red and gray areas between the red and black solid lines indicate the difference between the actual/measured densities and simulated equal distribution. A simulated Gaussian normal distribution with its peak density at the center (dashed line) and a simulated Gaussian normal distribution with its peak of the PSD or border of the PSD (dotted blue line). B1, B2) Two-dimensional density plot B1) and 1-dimensional radial distribution-analysis B2) as illustrated in A1) and A2) showing the distribution pattern of AMPA receptors at L4 spine PSDs. C1, C2) Two-dimensional density plot C1) and 1-dimensional radial distribution-analysis C2) as illustrated in A1) and A2) but for the distribution pattern of AMPA-receptor at L5 dendritic shaft PSDs. D1, D2) Two-dimensional density plot D1) and 1-dimensional radial distribution-analysis D2) as illustrated in A1) and A2) showing the distribution pattern of AMPA-receptors at L5 spine PSDs.

It is still rather unclear how neurotransmitter receptors are distributed at individual PSDs and whether they are arranged into clusters or distributed over the entire PSD. In addition, it also remains largely unknown whether the distribution pattern for a certain neurotransmitter receptor varies in relation to different layers of the neocortex. To address these questions normalized 2-dimensional receptor density maps (Figs. 9A1–D1 and 10A1–D1), and a 1-dimensional radial distribution-analysis (Figs. 9A2–D2 and 10A2–D2) were generated for each glutamate receptor type, layer, and target structure. The 2-dimensional receptor density maps allow the visualization of receptor density and distribution across all measured PSDs. Subsequent radial distribution-analysis allows the quantitative assessment of receptor distribution and the statistical comparison with simulated distributions. Both, in L4 and L5, AMPA-receptor density plots show a broad distribution across the PSDs.

**Fig. 10:**
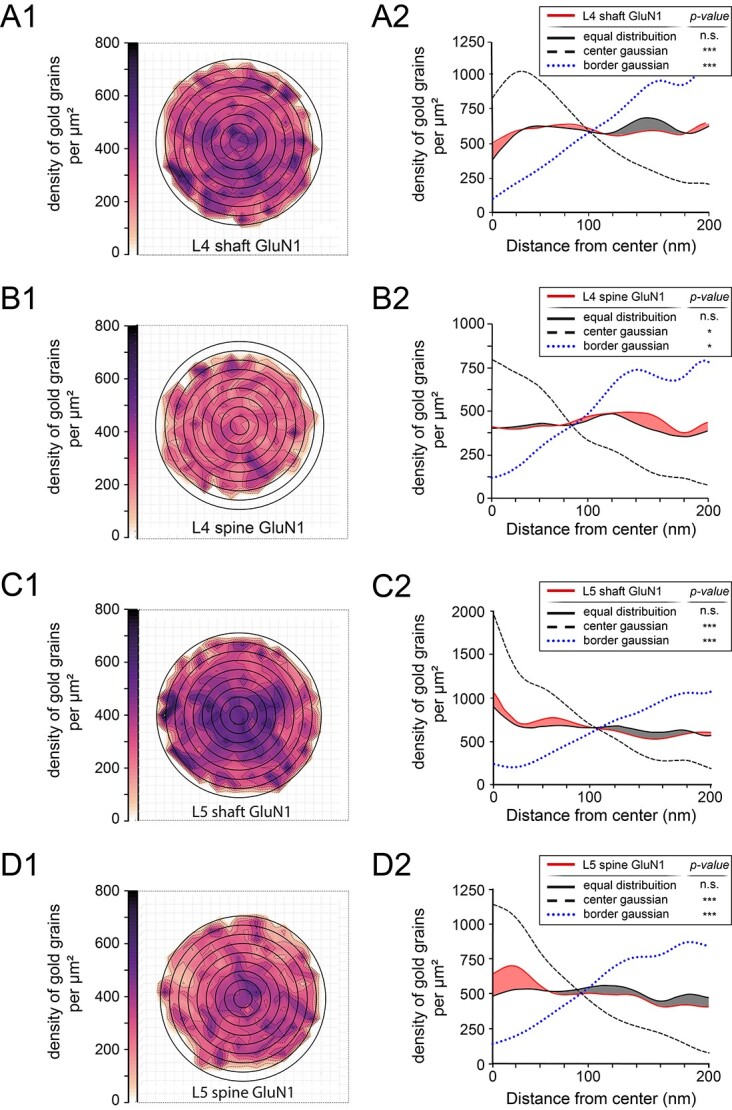
GluN1-subunit receptor distribution pattern at L4 and L5 PSDs. A1, A2) Two-dimensional density plot (A1) and 1-dimensional radial distribution-analysis A2) showing the distribution pattern of the GluN1-subunit of the NMDA-receptor at L4 shaft PSDs. B1, B2) Same 2-dimensional density plot B1) and 1-dimensional radial distribution-analysis B2) as illustrated in A1) and A2) for the GluN1-subunit of the NMDA-receptor at L4 spine PSDs. C1, C2) Two-dimensional density plot C1) and 1-dimensional radial distribution-analysis C2) as illustrated in A1 and A2) but for the GluN1-subunit of the NMDA-receptor at L5 shaft PSDs. D1, D2) Two-dimensional density plot D1) and 1-dimensional radial distribution-analysis D2) as illustrated in A1) and A2) showing the spatial distribution for the GluN1-subunit of the NMDA-receptor in L5 spine PSDs.

The highest AMPA-receptor density/μm^2^ was found in the central region for L4 spine and L5 dendritic shaft and spine PSDs (Fig. 9B1–D1), whereas for L4 dendritic shaft PSDs displayed a more homogeneous distribution (Fig. 9A1). Although AMPA-receptors occupied the central region of the PSDs, also areas of different shape, size, and intensities were found covering also the lateral edges of the PSD (Fig. 9A1–D1). In concordance with the generalized measurements (shown in Fig. 6), receptor density plots showed differences in the overall intensities (e.g. compare Fig. 9A1 vs. D1). Relative receptor distributions, however, appeared consistent across layers and target structures (e.g. compare Fig. 9B1 vs. D1).

To evaluate if receptor distributions are quantitatively and statistically homogeneously distributed, the dimensionality was reduced and a radial distribution analysis was performed (Fig. 9A2–D2). The measured datasets were compared with simulations of either a homogeneous equal distribution and heterogeneous center-Gaussian or border-Gaussian distribution (see Supplemental Fig. 2). Across both layers and target structures, center- and border-Gaussian distributions were significantly different to their corresponding measured datasets. No statistically significant difference, however, was found when comparing measured datasets with a simulated equal distribution. The latter supports the previously described impression of a more homogenous receptor distribution across the PSD surface.

For the GluN1-subunit of the NMDA-receptor, also the more central region of the PSD was occupied by the receptor for L4 and L5 dendritic shaft PSDs (Fig. 10A1 and C1) but weaker in density for L4 and L5 spine PSDs (Fig. 10B1 and D1). As already indicated by generalized receptor density measurements, overall intensities are found to be higher in shaft vs. spine target structures, and across both layers. Interestingly, the distribution pattern was comparable with the AMPA-receptor plots (Figs. 9 vs. 10). This finding was further supported by the absence of significant difference when comparing measured datasets with the simulated equal distribution (Fig. 10A2–D2). Simulated heterogeneous distributions showed significant differences across layers and target structures.

In summary, we can demonstrate that both receptors are quite similar distributed over the entire PSD but with differences in the receptor density and distribution pattern between layers and target structures (Figs. 9 and 10).

## Discussion

This is to our knowledge the first coherent and comprehensive quantitative study of the density and distribution pattern of AMPA- and NMDA-type glutamate receptors in the adult rat neocortex, exemplified in the barrel field of the somatosensory cortex. In L4 (main input layer of the neocortex) and L5 (main output layer of the neocortex) both AMPA- and the GluN1-subunit of the NMDA receptors showed layer- and target-specific differences (dendritic shaft vs. spine PSDs) in their density and distribution pattern. In addition, both receptors displayed a comparatively similar receptor distribution pattern at L4 and L5 dendritic and spine PSDs as visualized by receptor density maps. However, the highest receptor density was observed in the more central region of the PSDs with smaller sized spots to the PSD periphery. Strikingly, the AMPA/GluN1 ratio was largest at L4 and L5 spine PSDs suggesting beside a high synaptic efficacy also a strong modulatory effect in short-term plasticity.

Thus, both receptors, beside other structural factors, partially contribute to synaptic transmission, but also in the modulation of synaptic plasticity as observed in synaptically coupled pairs of neurons in the neocortex (see Discussion below). The comparatively high density of dendritic spines at L4 excitatory spiny neurons and L5 pyramidal cells together with the high excitability both glutamate receptor may also contribute to the stability of the columnar network.

### Quantitative analysis of excitatory neurotransmitter receptors in the neocortex

Meanwhile, numerous immunohistochemical studies have shown that glutamate receptors are found at neocortical synapses using either pre- or postembedding immunohistochemistry (see e.g. Kharazia and Weinberg 1997; He et al. 1998; Nusser 1999; Petralia et al. 1999; Petralia and Wenthold 1999; Gonzalez-Albo and DeFelipe 2000; He et al. 2000; Nusser 2000; Petralia et al. 2002; Petralia et al. 2003; Kooijmans et al. 2014; Lalanne et al. 2016; reviewed by Huntley et al. 1994; Wenthold et al. 2003; Sheng and Hoogenraad 2007; Fukazawa and Shigemoto 2012). However, these studies were more focused on the proof and abundance of a certain type of glutamate receptor and their possible co-localization at the PSD. Thus, coherent and comprehensive quantitative studies about their density and distribution are still relatively rare, in particular for individual layers of the neocortex in various animal species.

Receptor autoradiography, e.g. is widely used to generate region- and even layer-specific density maps in form of so-called “receptor fingerprints” of certain neurotransmitter receptors and their subunits in various brain regions and animal species but on a different scale (see e.g. Zilles and Palomero-Gallagher 2017; Palomero-Gallagher and Zilles 2019; Impieri et al. 2019; Palomero-Gallagher et al. 2020). The major advantage of this method is, beside its accuracy and reproducibility, the localization and distribution pattern of a certain receptor type as e.g. shown for the neocortex of wild type and reeler mice (Cremer et al. 2011). However, it does not allow the exact localization of these receptors at the prospective target structures at the cellular and subcellular level.

In contrast, pre- and postembedding immunohistochemical studies on ultrathin sections can visualize these receptors, their subunits and distribution at defined target structures at the cellular and subcellular level (see e.g. Nusser 1999; Petralia and Wenthold 1999; Nusser 2000; Kulik et al. 2002; Lopez-Bendito et al. 2002a, 2002b; Bergersen et al. 2008). However, in consecutive ultrathin sections covering the entire active zone, the immunolabeling often turns out unreliable due to methodological difficulties, because one could not expect reliable immunogold-labeling on consecutive ultrathin sections. Thus results obtained with this method are at least partially questionable with respect to a quantitative analysis. In addition, the need of permeabilization of biological membranes with surface-detergents does allow a penetration of the antibodies only to a depth of ~10 μm, but in most cases goes along with ultrastructural alterations that may affect the interpretation of the results.

However, two recent studies demonstrated a new method without the permeabilization (Fulton and Briggman 2021) or etching epoxy-resin-embedded ultrathin sections with Na-ethanolate (Holderith et al. 2020) both resulting in a high preservation of the ultrastructure together with a high-sensibility of antibody recognition at the EM level.

Nowadays FFRs combined with single- and multiple postimmunogold-labeling seems the method of choice for the quantitative analysis of various synaptic proteins, neurotransmitter receptor densities, and distribution pattern at the subcellular nanometer scale (see e.g. Fujimoto 1995; Hagiwara et al. 2005; Masugi-Tokita and Shigemoto 2007; Kasugai et al. 2010; Tabata et al. 2019; Nakamoto et al. 2020; reviewed by Takizawa and Robinson 2000; Fukazawa and Shigemoto 2012). The major advantage of FFRs combined with postimmunogold-labeling is 3-fold: First, in such preparations a carbon matrix of the surface membranes is highly preserved leading to a 2-dimensional image of biological tissues, in our case the unequivocal identification of IMPs constituting the PSD. Second, numerous PSDs in such preparations showed no distortions or malformations and thus can be regarded as intact structures that can be quantified. Finally, in all our single- and double labeling experiments whenever a PSD was present, also postimmunogold-labeling of a certain receptor type or both was observed to more than 98% accuracy. Hence, this approach is best suited for a quantitative analysis of the density and distribution pattern of neurotransmitter receptors as described here.

However, beside several advantages FRIL usually suffers from inherent problems, which are mainly associated with the large molecular size of the antibodies used. First, the use of primary and secondary gold-conjugated antibodies, the gold particles are usually ~20–30-nm away from the actual location of the antigen, thus preventing a precise detection of protein localization in biological membranes. Second, the bulkiness of antibody limits simultaneous multiple labeling of different components of a certain protein, or in our case the detection of several subunits of neurotransmitter receptors. Furthermore, the development of required N-terminal antibodies that are highly selective is often technically demanding.

Chemical protein labeling methods using small molecular probes could potentially overcome these problems. Tabata and co-workers (Tabata et al. 2019) developed a new peptide tag-probe pair for specific protein labeling and its application to EM detection of membrane proteins at the molecular level. This approach results in high labeling selectivity, enabling an improved EM detection of the labeled G-protein-coupled receptor in cell membrane of FFRs and in ultrathin sections. The efficiency and resolution obtained by the chemical labeling was significantly higher than those obtained by the immunogold-labeling were.

A recent study by Shigemoto and co-workers (Kleindienst et al. 2020) further developed a method to precisely measure the number and distribution of synaptic proteins. Here, FFRs immunogold EM was combined with a deep learning software “Darea,” analyzing replica images and demonstrated its usefulness for quick measurements of the pre- and postsynaptic areas, density, and distribution of gold particles at synapses in a reproducible and thus reliable automated manner.

However, further development of such methods would lead to an improved detection and visualization of, e.g. various synaptic proteins, neurotransmitter receptors, and their subunits at the subcellular molecular level.

### Contribution of glutamate receptors in L4 and L5 synaptic connections of the rat neocortex

At the molecular level, AMPA- and NMDA-type glutamate receptors are the major driving force of synaptic transmission as meanwhile shown by numerous paired- or multiple recordings of intra- and translaminar synaptic connections in the rodent neocortex (see e.g. Markram et al. 1997a, 1997b; Feldmeyer et al. 1999, 2002; Watanabe et al. 2005; Feldmeyer et al. 2006; Jiang et al. 2015; Rollenhagen et al. 2015; Qi and Feldmeyer 2016; Rollenhagen et al. 2018; Seeman et al. 2018; reviewed by Lübke and Feldmeyer 2007; Feldmeyer 2012). These studies suggest a layer- and target-specific differential contribution and recruitment of AMPA and NMDARs at depressing and facilitating synapses in the neocortex.

In L4–L4 excitatory spiny stellate (Feldmeyer et al. 1999; Seeman et al. 2018) and L5–L5 thick-tufted pyramidal cell synaptic connections (Markram et al. 1997a, 1997b; Rollenhagen et al. 2018; Seeman et al. 2018) in rodent and human neocortex showed marked differences in the reliability, synaptic efficacy, strength, short-term plasticity, and contribution of AMPA vs. NMDA receptors in synaptic transmission. The peak EPSP amplitude in L4–L4 excitatory connections was ~1.3-fold larger when compared with L5–L5 pyramidal cell connections. In some pairs of L4 spiny neurons, the unitary EPSPs were sufficiently large to elicit action potentials (APs) in the target neuron (Feldmeyer et al. 1999). This high efficacy was never observed in L5–L5 thick-tufted pyramidal cell connections. The percentage of failures of a presynaptic AP to elicit a unitary EPSP in L4–L4 connections was, on average, ~3-fold lower (5.3 ± 7.8% failures) when compared with L5 pyramidal cell pairs (14.3 ± 17.6%). Accordingly, the CV was larger in L5 connections (0.52 ± 0.37 vs. 0.37 ± 0.18). In addition, the latency, rise and decay time of the EPSPs may be affected by differences in the NMDA/AMPA-receptor ratios at these synapses as also shown in this study (Fig. 8). However, within each layer, the efficacy and strength could vary ~20-fold that can also be explained by our AMPA/GluN1 ratio analysis.

Glutamate receptors, as shown by our study, in excitatory spiny L4–L4 connections were of the AMPA and NMDA-type as revealed by pharmacological blockade experiments. At −60 mV in the presence of 1-mM Mg^2+^, NMDA receptors contributed 39.3 ± 12.5% to the EPSP integral. In Mg^2+^-free solution, the NMDA receptor/AMPA-receptor ratio of the EPSC was 0.86 ± 0.64. L5 excitatory connections were predominantly driven by AMPA-receptor type whereas NMDA-type receptors contributed ~20% to the unitary EPSP at −60 mV, but their contribution increased at more positive membrane potentials (Markram et al. 1997a; Rollenhagen et al. 2018). However, in both layers at individual synaptic connections also a large variability in the AMPA vs. NMDA ratio was observed.

In summary, the differential layer- and target-specific expression of both receptors investigated in this study partially contribute, beside other structural factors, to the functional differences in synaptic transmission between L4 and L5 synaptic connection, but may also explain differences in the paired pulse behavior at individual connections due to differences in the density and distribution pattern of both receptors and the AMPA/GluN1 ratio at individual PSDs.

### Postsynaptic effects related to the density and distribution pattern of AMPA- and NMDA-type glutamate receptors in the neocortex

Meanwhile, numerous studies showed that the differential expression and trafficking of neurotransmitter receptors at a given synapse directly affects their targeting to and retention within synaptic compartments and thus, the magnitude of synaptic transmission (see e.g. Kennedy and Ehlers 2006; Derkach et al. 2007; Lau and Zukin 2007; Shepherd and Huganir 2007; Kessels and Malinow 2009; Major et al. 2013; Matta et al. 2013; reviewed by Greger and Esteban 2007; Hansen et al. 2007; Rao and Finkbeiner 2007; Newpher and Ehlers 2008).

The majority of synaptic contacts (~80–85% depending on the brain region) on L4 spiny stellate cells and L5 pyramidal neurons are of the axo-spinous type, the remainder are axo-dendritic (~10–15%). In general, axo-spinous synaptic complexes are excitatory whereas axo-dendritic synapses are regarded as inhibitory, although there is evidence that not all dendritic shaft synapses are inhibitory (Silver et al. 2003). Although the functional relevance of dendritic spines is not fully understood in vitro and modeling experiments support the notion that: (i) spines substantially increase the surface area of a given dendritic segment to allow the establishment of a larger number of afferent synaptic contacts (Stepanyants et al. 2002); (ii) spines serve as individual biochemical and functional compartments by trafficking critical molecules such as various synaptic proteins or neurotransmitter receptors (reviewed by Hastings and Man 2018; Choquet and Hosy 2020); (iii) spines may play a regulatory role in the electrical properties of a neuron partially also driven by neurotransmitter receptors (Matsuzaki et al. 2004; Kopec et al. 2006; Araya 2014; Araya et al. 2014; reviewed by Sala and Segal 2014); (iv) spines may also contribute to coincidence EPSP/AP detection of backpropagating APs leading to either facilitation (Markram et al. 1997b) or depression (Egger et al. 1999), and (v) spines at terminal tuft dendrites of pyramidal neurons are the sites of evoked Ca^2+^-transients where glutamate receptors may also play a pivotal role in their induction, maintenance, and termination (Holbro et al. 2010).

PSDs are preferentially located at the spine head and are occupied by both AMPA- and NMDA-type glutamate receptors (see this study). Both receptors are linked to various signal cascades underlying efficacy and strength of synaptic transmission. However, structural and imaging studies have revealed the existence of various persistent and transient types of spines that differ substantially in shape and size suggesting differential functional roles in regulating time-scales for synaptic plasticity (Holtmaat et al. 2005, 2006). Remarkably, PSDs vary substantially in both shape and size at spines that upon stimulation undergo substantial structural changes (Matz et al. 2010; Holderith et al. 2012). In this study, the size of the PSD was well correlated with the number of both receptors/PSD suggesting differences in the functional properties of the EPSP and sensitization/desensitization kinetics at individual synaptic complexes depending on the number and ratio of both receptors and their availability at a given PSD. On average, the density of AMPA-receptors in L4 and L5 is quite similar and comparably high on dendritic spines vs. dendritic shafts, suggesting a strong reliability in synaptic transmission at spines. This is in line with observations using paired recordings (Markram et al. 1997a, 1997b; Feldmeyer et al. 1999, 2002; Seeman et al. 2018). Strikingly, the density of the GluN1-subunit of the NMDA-receptor is higher at dendritic shafts in L4 and L5 pointing to a more modulatory and prolonged kinetics of the EPSP amplitude in L4 and L5 excitatory connections. However, the density in the distribution of both receptor values showed a large variability as indicated by the large SD and CV that may also contribute to differences in the paired-pulse behavior at individual synaptic complexes.

The high density of axo-spinous synapses along the dendritic tree of L4 spiny stellate and L5 pyramidal neurons and the comparably large PSDs containing a relatively high number of both types of receptors may also contribute to the temporal-timed co-incidence detection of backpropagating APs. Depending on the AP/EPSP coincidence at a small 10-ms time window (L5 excitatory connections) at spines lead to either facilitation (L5; Markram et al. 1997b) or at a time window of 25 ms to depression (L4; Egger et al. 1999). However, in L4, depression is induced by metabotropic group II and not by AMPA and NMDA receptors (Egger et al. 1999).

In summary, the high density of excitatory axo-spinous vs. axo-dendritic synaptic complexes together with the high density and distribution pattern of both glutamate receptors at PSDs at L4 spiny stellate and L5 pyramidal cell dendrites contribute to the observed high synaptic efficacy and strength in synaptic transmission induced by AMPA-receptors but also a strong modulation of short-term plasticity driven by NMDA-receptors.

### Glutamate receptors and cortical connectivity

How can glutamate receptors contribute to cortical connectivity? Spiny stellate and star pyramidal neurons in L4 represent the major input station of signals from the sensory periphery via the respective thalamic relay nuclei (reviewed by Sherman 2012). Thus, L4 represents the first station of intracortical information processing. It has been demonstrated that excitatory L4 neurons are capable to enhance even weak thalamic signals (Bruno and Sakmann 2006), can generate postsynaptic APs and were thus regarded as “cortical amplifiers” (Feldmeyer et al. 1999) within the circuitry of the cortical column. The connectivity between spiny stellate neurons in L4 (~3,400 neurons/barrel) in rat barrel cortex is comparatively high; each L4 neuron is interconnected with ~ 200–250 other spiny stellate neurons and synaptic contacts were predominantly (~95%) established on dendritic spines. In addition, an individual L4 neuron innervates ~ 300–400 L2/3 pyramidal neurons and ~300–400 L4 spiny stellate neurons synapse on a single L2/3 neuron (Lübke et al. 2003; Feldmeyer et al. 2006). Furthermore, L4 spiny neurons are interconnected with L5 (Feldmeyer and Sakmann 2000; Feldmeyer et al. 2005) and L6 (Qi and Feldmeyer 2016). Hence, the function of L4 excitatory spiny neurons is to reliably transmit thalamic and intracortical signals throughout the canonical circuit of the cortical column.

Taken together, several electrophysiological properties (large EPSP amplitudes, high release probability, and low failure rates; Feldmeyer et al. 1999, 2002) and structural factors (large PSDs, large readily releasable pools; Rollenhagen et al. 2015), the high density of synaptic spines containing a relatively high number of AMPA and NMDA-type of glutamate receptors all contribute to the high connectivity of L4 spiny neurons and their respective target neurons. It can be speculated that the relatively “high” density of AMPA- and NMDA-receptors at L4 PSDs help to sustain excitability (Choquet 2018) and “stability” of the columnar network, but may also help to sharpen the network properties in L4 itself, but also in L2/3, L5, and L6 pyramidal neurons with which L4 neurons are interconnected. Furthermore, it has been demonstrated that excitation (Holtmaat et al. 2005, 2006) and neurotransmitter receptors (Passafaro et al. 2003) promotes the emergence of newly generated “functional” spines and as a consequence the regulated internalization of AMPA- and NMDA-type glutamate receptors to the PSD that further contribute to the stabilization of the columnar network.

Whereas L4 excitatory neurons represent the input station of the neocortex and “serve” to intracolumnar signal transduction and processing, L5 pyramidal cells are the main output station of the neocortex to the contralateral hemisphere via the corpus callosum and to various subcortical brain regions (reviewed by Ramaswamy and Markram 2015). Beside intracolumnar information processing via their vertical ascending axonal collaterals, L5 pyramidal neurons via their long-range horizontal axonal collaterals are interconnected with L5 pyramidal neurons across columns and may thus serve as “integrators” across cortical columns (reviewed by Ramaswamy and Markram 2015). Taken that a cortical column is ~ 300 μm in widths, together with connection probability of 10–15% and a potential of 5 synaptic contacts/connection, a single L5 pyramidal neuron is innervated by ~40 neighboring pyramidal neurons receiving about 200 afferent synaptic inputs (Ramaswamy et al. 2012). It may be speculated that due to the high density of dendritic spines, large PSDs with a “high” number of AMPA and NMDA-type glutamate receptors they are involved not only in the induction, maintenance and termination of synaptic transmission but also in reliability and maintenance of the intracolumnar, transcolumnar, and extracortical network in which L5 pyramidal neurons are embedded.

### Future directions

Here, we have described the density and distribution pattern of 2 glutamate receptors in the input layer L4 and the output layer L5 of the neocortex. It would be interesting to know how both receptors are organized in other layers of the neocortex constituting the cortical column, the fundamental building block of the neocortex. Also, quantitative data about other major neurotransmitter systems in the neocortex at the cellular and subcellular EM level are still rare or not available. Second, what is the scenario of neurotransmitter density and distribution in a pathologically altered neurodegenerative or neurological neocortex? Finally, one important question in synaptic neuroscience is whether findings in experimental animals can be one-to-one transferred into the human brain. Hence, we have started to work on neurotransmitter receptors in the human temporal lobe neocortex using both nonaffected and affected biopsy material taken during epilepsy surgery to analyze and compare the possible co-localization, density, and distribution pattern of various neurotransmitter receptors in the normal and pathologically altered human brain (work in preparation).

## Supplementary Material

Suppl_Figure_1_bhac212

Suppl_Figure_2_bhac212

Receptor_paper_Supp_Fig_Legends_bhac212
