# Supplementary figures and images for: Layer-specific distribution and expression pattern of AMPA- and NMDA-type glutamate receptors in the barrel field of the adult rat somatosensory cortex: a quantitative electron microscopic analysis
Source: Cereb Cortex. 2022 Jun 21;33(5):2342–60. doi: 10.1093/cercor/bhac212 (PMC9977369; doi:10.1093/cercor/bhac212)

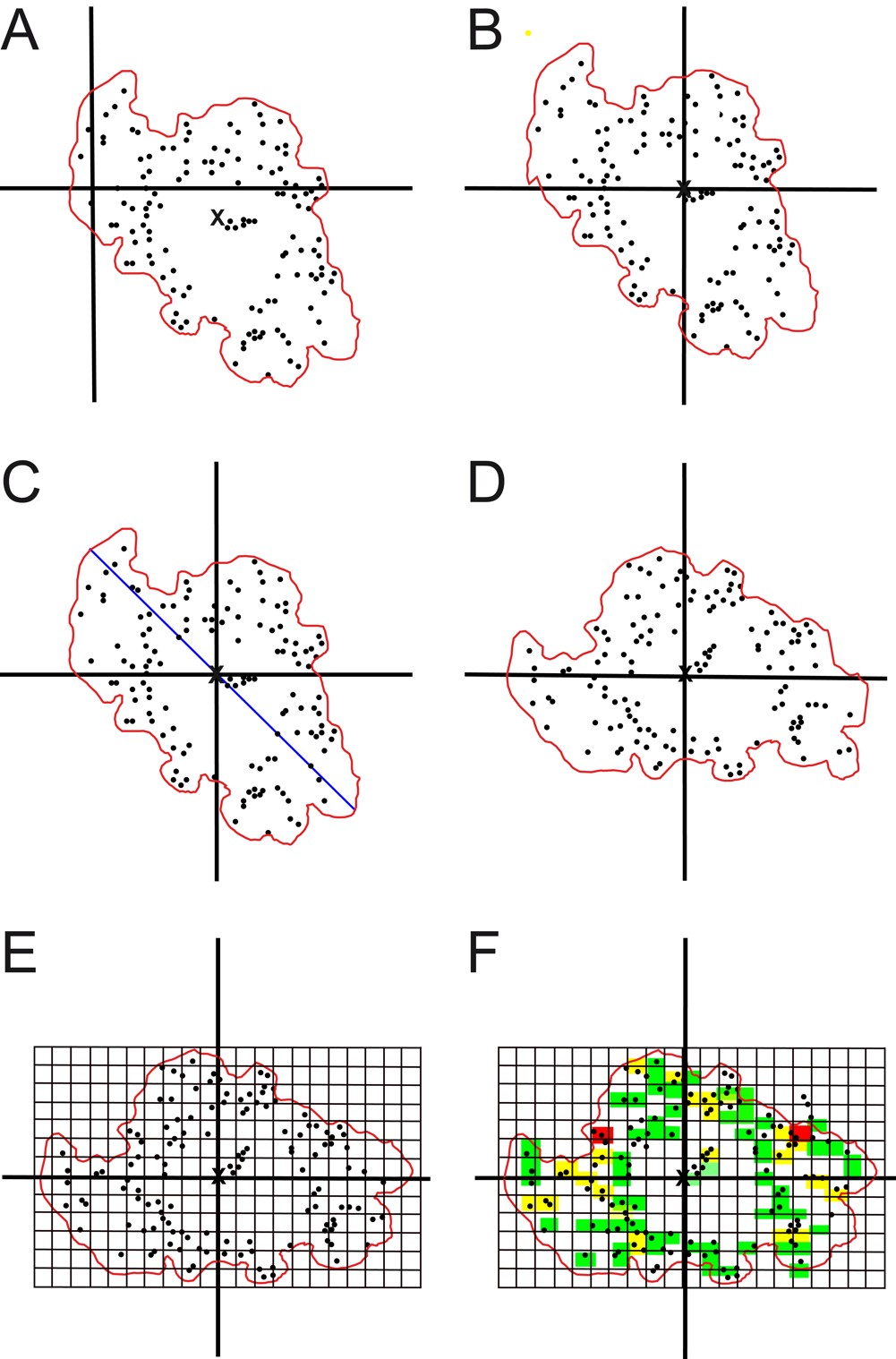

Supplement: Suppl_Figure_1_bhac212 [file suppl_figure_1_bhac212.jpeg]

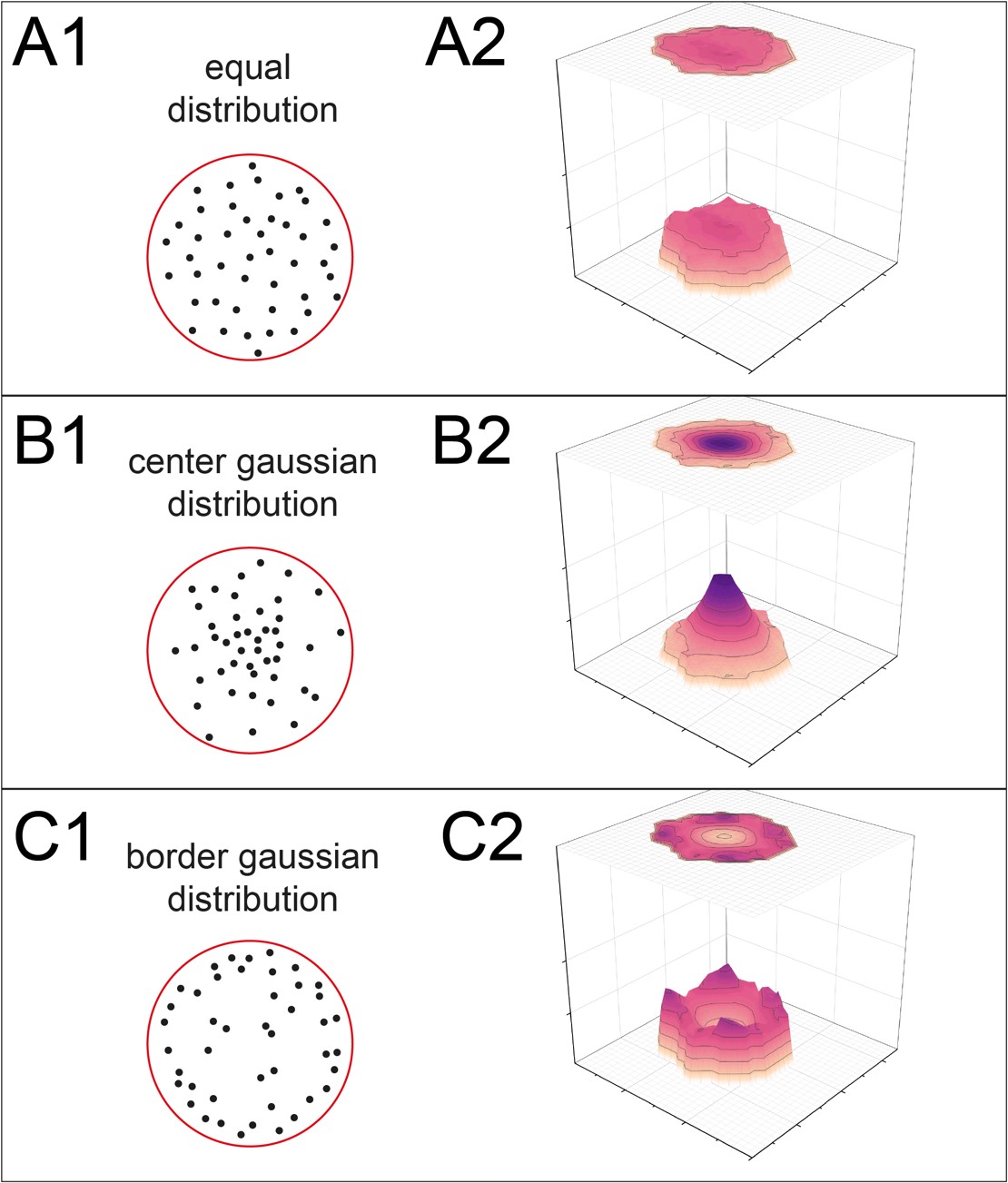

Supplement: Suppl_Figure_2_bhac212 [file suppl_figure_2_bhac212.jpeg]
