# Supplementary material for: Layer-specific distribution and expression pattern of AMPA- and NMDA-type glutamate receptors in the barrel field of the adult rat somatosensory cortex: a quantitative electron microscopic analysis
Source: Cereb Cortex. 2022 Jun 21;33(5):2342–60. doi: 10.1093/cercor/bhac212 (PMC9977369; doi:10.1093/cercor/bhac212)
Supplement: Receptor_paper_Supp_Fig_Legends_bhac212 [file receptor_paper_supp_fig_legends_bhac212.docx]

**Supplemental Fig. 1: Generation of receptor distribution maps**

**A,** After contouring the PSD and estimation of the number of gold grains a center of gravity was calculated.

**B,** Alignment of the PSD to the center of gravity.

**C,** Calculation of the maximal diameter of the PSD (blue line).

**D,** Rotation of the PSD to orient the maximal diameter to the horizontal axis.

**E,** Rasterization of the PSD by placing a grid field of known dimension.

**F,** Creation of a receptor density map.

**Supplemental Fig. 2: Simulation of receptor distribution maps**

**A1,** Schematic illustration of a random equal distribution. Red circle illustrates the PSD outline, black dots illustrate the receptors.

**A2,** Example of a 3D density plot with a simulated random equal distribution. 2D density plot on top shows the color-coded projection of the X-Y plane.

**B1,** Schematic illustration of a center gaussian distribution. Red circle illustrates the PSD outline, black dots illustrate the receptors.

**B2,** Example of a 3D density plot with a simulated center gaussian distribution. 2D density plot on top shows the color-coded projection of the X-Y plane.

**C1,** Schematic illustration of a border gaussian distribution. Red circle illustrates the PSD outline, black dots illustrate the receptors.

**C2,** Example of a 3D density plot with a simulated border gaussian distribution. 2D density plot on top shows the color-coded projection of the X-Y plane.
